# Supplementary material for: Content-rich biological network constructed by mining PubMed abstracts
Source: BMC Bioinformatics. 2004 Oct 8;5:147. doi: 10.1186/1471-2105-5-147 (PMC528731; doi:10.1186/1471-2105-5-147)
Supplement: Additional File 2 — The original results of the above study (non-essential files are deleted to keep the file size under the limit set by BMC bioinformatics). [file 1471-2105-5-147-S2.bz2 › chilibotAdditionalFile2/dip20/371ID10950949E6046/html/FGF2_FGFR1.html]

 


 **FGF2** and **FGFR1** 
  
Found 575 abstracts in PubMed, retrieved 20.  
 

 What does Google say? 
 PDF only 
| .edu only 

---

**Interactive relationship** (e.g. stimulation, inhibition, etc)

**Stimulatory relationship**- In situ hybridization analyses of TRalpha knockout mice, which have impaired ossification and skeletal mineralization, revealed reduced  **FGFR1**  mRNA expression in osteoblasts and osteocytes, whilst T3 failed to stimulate  **FGFR1**  mRNA or enhance  **FGF2**  activated MAPK signaling in TRalpha null osteoblasts.  Ref: 12805413 Mol Endocrinol< MedlineTA>Mol Endocrinol,
- Analysis of tryptic phosphopeptide maps of FGFR 1  [ **FGFR1** ]  from cells stimulated with FGF 2  [ **FGF2** ]  alone and FGF 2  [ **FGF2** ]  together with heparin showed that FGF 2  [ **FGF2** ]  alone stimulated a several fold increase in tyrosine 463 in the juxtamembrane domain.  Ref: 12799194 Exp Cell Res, 2003
- Hence, FGF 2  [ **FGF2** ]  attached to the heparan sulfate moiety expressed by the novel CD44 variant of RA synovium cells exhibits an augmented ability to stimulate FGFR 1  [ **FGFR1** ]  mediated activities.  Ref: 12697740 J Clin Invest, 2003
**Neutral relationship**- We find that SMC and BAEC express the same FGFR  [ **FGFR1** ]  isoforms and bind  **FGF2**  with equal affinity at the cell surface, yet FGF has a markedly higher proliferative effect on SMC than on BAEC.  Ref: 12659634 Biochem J< MedlineTA>Biochem J, 2003
- Our studies have shown that full length FGF receptor 1  **FGFR1**  accumulates within the nuclear interior in parallel with FGF 2  [ **FGF2** ] .  Ref: 12647309 J Cell Biochem, 2003
- However, Namalwa cells, immobilizing FGF 2  [ **FGF2** ]  via their cell surface CD44vRA, bound substantially more soluble FGF receptor 1 FGFR 1  [ **FGFR1** ]  than did Namalwa cells immobilizing the same amount of FGF 2  [ **FGF2** ]  via their cell surface CD44v3 v10.  Ref: 12697740 J Clin Invest, 2003
- The biological activity of bFGF  [ **FGF2** ]  is mediated through interaction with its high affinity receptor, fibroblast growth factor receptor 1  [ **FGFR1** ]  FGFR 1  [ **FGFR1** ] .  Ref: 12651849 J Biol Chem< MedlineTA>J Biol Chem, 2003
- Exogenous FGF 2  [ **FGF2** ]  decreased  **FGFR1**  protein.  Ref: 12746216 Am J Physiol Endocrinol Metab< MedlineTA>Am J Physiol Endocrinol Metab, 2003

**Non-interactive relationship** (e.g. studied together, co-existance, homology, etc.)

- The objective of this study was to elucidate the correlation between the expression of FGF 2  [ **FGF2** ] , VEGF, and their receptors FGF receptor 1 FGFR 1  [ **FGFR1** ]  and VEGF receptor 1 Flt 1 with microvessel density in synovial tissues of the TMJ.  Ref: 12651930 J Dent Res, 2003
- METHODS The expression of VEGF, FLK 1, bFGF  [ **FGF2** ]  and FLG  [ **FGFR1** ]  in 40 young and 30 postmenopausal women with breast cancer was studied by immunohistochemical method SABC, with its relation with axillary lymph node metastasis and the clinical and pathologic characteristics.  Ref: 12795839 Zhonghua Zhong Liu Za Zhi, 2003
- Colocalisation of  **FGFR1**  with FGF 2  [ **FGF2** ]  could not be demonstrated in unstressed retina, but was demonstrable in stressed retina, in both the ONL and OPL.  Ref: 12708794 Growth Factors, 2002
- METHODS Immunohistochemistry and confocal microscopy were used to localise FGF 2  [ **FGF2** ]  and  **FGFR1**  in cryosections of the rat retina, both normal retina and retina stressed by exposure to bright continuous light 1000 lux, 24h.  Ref: 12708794 Growth Factors, 2002
- Sensitivity to FGF 2  [ **FGF2** ]  disappeared after 7 days although  **FGFR1**  expression was maintained.  Ref: 12746216 Am J Physiol Endocrinol Metab< MedlineTA>Am J Physiol Endocrinol Metab, 2002
- We conclude that TSH stimulates  **FGFR1**  but not FGF 2  [ **FGF2** ]  expression.  Ref: 12746216 Am J Physiol Endocrinol Metab< MedlineTA>Am J Physiol Endocrinol Metab, 2002
- We also measured components of the FGF 2  [ **FGF2** ]  ternary signaling complex, fibroblast growth factor receptor 1  [ **FGFR1** ]  FGFR 1  [ **FGFR1** ]  and syndecan 4.  Ref: 12832283 Am J Physiol Lung Cell Mol Physiol< MedlineTA>Am J Physiol Lung Cell Mol Physiol, 2002
- Similarly, T3 enhanced  **FGF2**  activated autophosphorylation of  **FGFR1** , but did not modify  **FGF2**  induced phosphorylation of the docking protein FRS2.  Ref: 12805413 Mol Endocrinol< MedlineTA>Mol Endocrinol, 2002
- There was also a significant difference in VEGF, bFGF  [ **FGF2** ] , FLK 1, FLG  [ **FGFR1** ]  and MVC between the stage 0 approximately II and stage III approximately IV P <.  Ref: 12795839 Zhonghua Zhong Liu Za Zhi, 2003
- FGF 2  [ **FGF2** ]  alone stimulated maximal phosphorylation of Src homology domain 2 tyrosine phosphatase SHP 2 and the adaptor molecule Crk, in heparan sulfate deficient Chinese hamster ovary CHO 677 cells expressing FGFR 1  [ **FGFR1** ] .  Ref: 12799194 Exp Cell Res, 2003
- The mean expression of VEGF, bFGF  [ **FGF2** ] , FLK 1 and FLG  [ **FGFR1** ]  of axillary lymph node positive patients was higher than the negative ones both in young and postmenopausal women groups P <.  Ref: 12795839 Zhonghua Zhong Liu Za Zhi, 2003
- noggin expression is suppressed by  **FGF2**  and syndromic fgfr  [ **FGFR1** ]  signalling.  Ref: 12687003 Nature, 2003
- Thyroidal levels of fibroblast growth factor 2  [ **FGF2** ]  FGF 2  [ **FGF2** ]  and fibroblast growth factor receptor 1  [ **FGFR1** ]   **FGFR1**  are elevated in human thyroid hyperplasia.  Ref: 12746216 Am J Physiol Endocrinol Metab< MedlineTA>Am J Physiol Endocrinol Metab, 2003
- Colocalisation of  **FGFR1**  and FGF 2  [ **FGF2** ]  could be demonstrated in stressed retina, in the cytoplasm and the axon terminals of photoreceptors.  Ref: 12708794 Growth Factors, 2002
- To understand the significance of this, effects of  **FGFR1**  activation on normal human thyrocyte growth and function in vitro and the regulation of FGF 2  [ **FGF2** ]  and  **FGFR1**  expression have been examined.  Ref: 12746216 Am J Physiol Endocrinol Metab< MedlineTA>Am J Physiol Endocrinol Metab, 2002
- Increases in  **FGFR1**  or FGF 2  [ **FGF2** ]  and both may contribute to goitrogenesis.  Ref: 12746216 Am J Physiol Endocrinol Metab< MedlineTA>Am J Physiol Endocrinol Metab, 2002
- We have studied the mechanism whereby heparin directs FGF 2  [ **FGF2** ]  induced FGF receptor 1 FGFR 1  [ **FGFR1** ]  signal transduction.  Ref: 12799194 Exp Cell Res, 2003
- AIM To identify sites of FGF 2  [ **FGF2** ]  action on photoreceptors of the rat retina, by localizing  **FGFR1**  in the intact retina, and to assess the colocalisation of FGF 2  [ **FGF2** ]  with  **FGFR1** .  Ref: 12708794 Growth Factors, 2002
- Immunohistochemical assays for bFGF  [ **FGF2** ] , fibroblast growth factor receptor 1  [ **FGFR1** ]   **FGFR1** , and proliferating cell nuclear antigen were performed.  Ref: 12784342 Cancer, 2003
- Heparan sulfate glycosaminoglycans derived from endothelial cells and smooth muscle cells differentially modulate Fibroblast Growth Factor 2  [ **FGF2** ]  biological activity through Fibroblast Growth Factor Receptor 1  [ **FGFR1** ] .  Ref: 12659634 Biochem J< MedlineTA>Biochem J, 2003
- These changes are associated with specific alterations in the regulation of FGF 2  [ **FGF2** ] , FGFR 1  [ **FGFR1** ]  and syndecan 4 in the airway epithelial mesenchymal trophic unit, which may be associated with the developmental problems of the lung associated with exposure to ozone.  Ref: 12832283 Am J Physiol Lung Cell Mol Physiol< MedlineTA>Am J Physiol Lung Cell Mol Physiol, 2003
- Finally, isolated primary synovial fluid cells from RA patients expressing CD44vRA bound more soluble FGFR 1  [ **FGFR1** ]  to their cell surface associated FGF 2  [ **FGF2** ]  than did corresponding synovial cells expressing CD44v3 v10 or synovial cells from osteoarthritis patients.  Ref: 12697740 J Clin Invest, 2003
- **FGFR1**  expression and  **FGFR1**  FGF 2  [ **FGF2** ]  colocalisation in rat retina sites of FGF 2  [ **FGF2** ]  action on rat photoreceptors.  Ref: 12708794 Growth Factors, 2002
